# Validity and reliability of the Chinese version of the Break-up Distress Scale for college students

**DOI:** 10.3389/fpsyg.2025.1615021

**Published:** 2025-07-02

**Authors:** Xin Qiao, Tingting Zhan, Yaoguo Geng

**Affiliations:** ^1^School of Marxism, Zhengzhou University, Zhengzhou, China; ^2^School of Marxism, Shenyang Aerospace University, Shenyang, China

**Keywords:** break-up distress, reliability, validity, college students, scale

## Abstract

**Objective:**

This study aimed to revise the validity and reliability of the Chinese version of the Break-up Distress Scale (BDS) for college students.

**Methods:**

A total of 669 college students with breakup experience in the last 6 months were selected using the online survey platform Questionnaire Star and randomly divided into two groups: Sample 1 for exploratory factor analysis (EFA) and Sample 2 for confirmatory factor analysis (CFA). Depression, anxiety, and sleep subscales of the DSM-5 Self-Rated Level 1 Cross-Cutting Symptom Measure-Adult were used as criterion tools.

**Results:**

The EFA supported the one-dimensional model of the original scale. The CFA showed that the model structure fit well (χ^2^/*df* = 1.792, *GFI* = 0.935, *AGFI* = 0.915, *NFI* = 0.958, *RMSEA* = 0.048). *Cronbach’s α* of the BDS was 0.964, and the total score of the scale was significantly and positively correlated with the criterion scales. The scale demonstrated strict equivalence across genders and breakup initiators. Males’ scores were significantly higher than those of females, and the break-up distress scores of breakup initiators were significantly lower than those of dumped people.

**Conclusion:**

The Chinese version of the BDS can be used to assess distress in Chinese college students. The scale has satisfactory reliability and validity.

## 1 Instruction

Engaging in romantic relationships is prevalent among college students (Brassard et al., 2018; Dooley et al., 2015). Statistical surveys among Chinese college students indicated that 63.6% of college students were either in a romantic relationship or had experienced one in the past; however, the problem of breakups was increasingly prominent, with 54.7% of students having undergone a breakup. In recent years, within the scope of psychological counseling services provided at universities, consultations associated with romantic relationships have accounted for 37.68% of the total (Wang and Zhang, 2011). Distress experienced after the dissolution of a romantic relationship is referred to as break-up distress, which includes a series of maladaptive reactions. For example, repeatedly immersing oneself in recollections of past relationships (Harake and Dunlop, 2020), continuously experiencing negative emotions such as sadness and anxiety (Field et al., 2009; Sprecher, 1994), and showing behavioral characteristics such as social withdrawal and a decrease in interpersonal trust in social interactions (Geng et al., 2023). These experiences are characterized by both intrinsic, intense discomfort and sustained negative experiences of frustration, which can exacerbate the crisis that threatens an individual’s stability, potentially leading to a collapse (Dooley et al., 2015). Consequently, researchers have identified college students who have experienced a breakup as one of the five major categories in the realm of crisis psychology among college students (Duan and Cheng, 2006). Hence, it is imperative to evaluate the extent of the break-up distress experienced by college students.

Break-up distress refers to the negative emotions (e.g., depression, anxiety, stress, guilt, remorse, and anger) experienced by individuals after the dissolution of romantic relationships, as well as a series of behavioral problems such as insomnia and eating disorders (Boelen and Reijntjes, 2009; Brassard et al., 2018; Field et al., 2009). Based on previous research, this study operationally defines break-up distress as a multidimensional state of cognitive-emotional-behavioral dysfunction following the termination of an intimate relationship. Break-up distress is characterized by obsessive rumination about romantic relationships and breakup experiences, sustained activation of negative emotions (e.g., pain and anger), and temporary impairment of social functions (e.g., decreased interpersonal trust). Unlike stress responses caused by other traumatic events, break-up distress represents a short-term adjustment disorder after the dissolution of a romantic relationship, focusing on the specific reactions to the “loss of intimacy.” In short, break-up distress refers to physical or psychological distress caused by the dissolution of a romantic relationship.

Several assessment measures for break-up distress primarily aim to evaluate emotional experiences following a split. For instance, Sprecher (1994) used a 7-point response scale composed of negative and positive emotions and *Emotional Reactions after the Breakup* to assess the affective experiences of 47 young couples after their breakup. Ultimately, 14 emotions were selected to evaluate break-up distress and categorized into *nine negative emotions* (depression, guilt, resentment, anger, frustration, hatred, loneliness, injury, and jealousy) and *five positive emotions* (love, satisfaction, contentment, happiness, and liberation). In addition, the existing assessment tools for break-up distress abroad are mostly derived from the *Complex Grief Inventory* (ICG) developed by Prigerson et al. (1995). The ICG was originally designed to measure a series of maladaptive symptoms produced by the experience of grief following the loss of a loved one. The dissolution of romantic relationships represents a type of loss that individuals experience in intimate relationships, similar to the loss brought by the death of relatives (Xiao and Xiaoming, 2018). Boelen et al. (2003) revised the ICG to create a Dutch version of the Inventory of Traumatic Grief. Field et al. (2009) revised the ICG to develop the *Break-up Distress Scale* (BDS) for a college student sample, replacing descriptions associated with the loss of a loved one with those related to a breakup, such as revising “I have difficulty believing in others since he/she died” to “I have difficulty believing in others since the breakup.” In addition, three questions were deleted during the revision process (“I have heard the person who died speaking to me,” “I have seen the person who died standing in front of me,” and “I feel it is unfair that I am still alive while the other person is gone”), resulting in a final BDS with 16 questions. Studies have shown that the BDS has good reliability and validity and is widely used to assess the degree of break-up distress experienced by college students (Brewer and Abell, 2017; Bronfman et al., 2016; Field et al., 2010; Norona et al., 2018).

Gender differences and breakup initiators are popular topics in the dissolution of romantic relationships (Johnson et al., 2024). Research has shown that males and females differ in their emotion regulation methods, utilization of social support, and perception of relationships when facing breakups. For example, males may be more inclined to suppress their emotions, whereas females are better at alleviating pain by talking. These differences may lead to variations in the degree of break-up distress (Geng et al., 2023). In addition, females tend to adopt more life restoration-oriented strategies after the dissolution of romantic relationships, which indicate as continuing daily life habits and seeking new ways to distract and motivate themselves. However, males tend to adopt more loss-oriented strategies, such as immersing themselves in the distress of dissolution, having an urge to cry, and ruminating on the negative emotions brought by the break-up (Sánchez-Porro and Silva-Vicuña, 2024). Regarding the identity of the breakup initiator, there were also significant differences in the psychological experiences between the individuals who initiated the breakup and those who passively accepted it. Those who initiated the breakup may have undergone certain psychological adjustments before the dissolution of their romantic relationships. They experienced an improvement in their subjective well-being and saw benefits in other areas of life, with the exception of the economic domain. However, those who passively accept the breakup may experience more negative emotions, such as a sense of betrayal, anger, and rejection. They experience a significant short-term loss in subjective well-being and a high level of break-up distress (Brüning, 2022). This study examines the effect of gender and breakup initiators on break-up distress. While replicating the results of previous studies, this study verified the validity of the Chinese version of the BDS.

In recent years, break-up distress has emerged as a primary trigger of maladaptive behaviors in early adulthood, garnering attention from researchers. Research indicates that all Chinese undergraduate students who have experienced a breakup feel a strong sense of shame along with emotional confusion, internal despair, anger, and depression (Zeng et al., 2022). In addition, break-up distress is significantly associated with low life satisfaction (Rhoades et al., 2011). Thus, the negative impact of breakup pain on the mental health of Chinese college students cannot be underestimated. However, there is a lack of specific tools to assess break-up distress in China. Some studies use the Symptom Check List 90 (SCL-90), a tool mainly used for the assessment of psychiatric symptoms (Cui et al., 2014), to assess the breakup emotions of college students, which limits research on break-up distress to a certain extent (Wang and Zhang, 2011). In addition, as part of a social group, college students, under the influence of the mainstream culture of society, consciously or unconsciously imprint their views on love with the imprint of traditional Chinese culture (Huang and Zheng, 2012). Specifically, phrases with high cultural resonance and recognition, such as “Hold your hand and grow old together” and “Wish to have one’s heart and stay together until old age,” clearly reflect college students’ profound longing and idealized pursuit of eternal love (Zhang et al., 2023). Therefore, the connotations and expressions of pain associated with breakups among Chinese college students exhibit distinct characteristics. Therefore, the development of convenient and appropriate assessment tools is necessary.

This study fully considered the above situation, revised the BDS, and tested its reliability and validity to provide an effective measurement tool for research on break-up distress among college students. The application of this scale to Chinese college students who break up can provide more scientific, effective, and extensive measurement data and support for mental health monitoring, psychological counseling, and related research.

## 2 Methods

### 2.1 Participants

Questionnaires were distributed through an online platform to participants who had experienced a breakup in the past 6 months. Participants were asked to complete the BDS and criterion measures. After removing the questionnaires that were completed quickly, 675 questionnaires were distributed. The IP addresses of the participants covered 20 provincial administrative regions across China, demonstrating that the sample data of this study exhibit a certain level of representativeness. In total, 669 valid questionnaires were collected. The study sample consisted of 284 males and 385 females. There were 285 individuals who were only children and 384 who were not. The age of participants ranged from 18 to 26 years old (2.38 ± 4.42). Of the participants, 188 reported initiating the breakup themselves, whereas 186 reported being at the receiving end of the breakup (Table 1).

**Table 1:** General demographic information of participants.

Variable	Group	Number	Percent (%)
Gender	Male	284	42.5
	Female	385	57.5
Only children or not	Only children	285	41.9
	Not children	384	56.5
Breakup initiator	Self	188	28.1
	Partner	186	27.8
	Both	130	19.4
	Neither	105	15.7
	Unclear	60	9.0
Number of dating relationships	Once	206	30.8
	Twice	204	30.5
	Three times	115	17.2
	Four or more times	144	21.5

### 2.2 Measures

#### 2.2.1 Break-up distress

The BDS is a revised version of the ICG developed by Field et al. (2009) and Prigerson et al. (1995). It comprises 16 items to assess the level of distress associated with a breakup. The scale ranges from 1 (not at all) to 4 (very much). The original scale had a Cronbach’s *α* of 0.94, indicating high internal consistency and reliability.

After obtaining authorization from the original scale compiler, the scale was revised. The scale was translated from English to Chinese following Brislin’s back-translation protocol (Brislin, 1970). First, a professor majoring in psychology translated the scale into Chinese according to the Chinese cultural background and expressions, and the preliminary Chinese version of the scale was formed through concentrated discussion. Two English professors translated the Chinese version of the scale and modified the scale again according to the translated results. The Chinese version of the Breakup Pain Scale was obtained after the revised scale was translated and modified. Second, 13 college students (six males and seven females) were selected for the initial scale measurement. Interviews were conducted after the initial test to ensure that all the items on the scale were understandable and clear. Finally, a panel of three professional psychologists discussed the final scale. The Chinese version of the BDS uses a 4-point score (1 = not at all, 4 = completely). There are 16 items, and the total score is used as the evaluation index. That is, the higher the total score, the higher the level of break-up distress. The Chinese and English version of BDS can be found in the Appendix.

#### 2.2.2 DSM-5 self-rated level 1 cross-cutting symptom measure-adult

The DSM-5 Self-Rated Level 1 Cross-Cutting Symptom Measure-Adult is a scale compiled by Narrow et al. (2013) based on the Diagnostic and Statistical Manual of Mental Disorders, Fifth Edition (DSM-5), which was later revised by Chinese researchers such as Zhu et al. (2019). The scale consists of 23 items that evaluate specific symptoms for the past 2 weeks. Research has shown that individuals experience negative emotions, such as depression and anxiety, after the end of a romantic relationship, along with varying degrees of sleep problems (Boelen and Reijntjes, 2009). Therefore, the current study selected the depression, anxiety, and sleep subscales as the criterion scales, which consisted of six items. The *depression* subscale included two items (e.g., having little or no interest in or pleasure in doing things), the *anxiety* subscale included three items (e.g., feeling panic or fear), and the *sleep* subscale included one item (e.g., sleep problems affecting overall sleep quality). The scale uses a 5-point score, with 0 indicating “none” or “not at all” and 4 indicating “severe” or “nearly every day,” with higher scores indicating more severe symptoms. In the current study, Cronbach’s *α* for this scale was 0.955.

### 2.3 Procedure

All the participants provided informed consent electronically through a secure online platform. The consent form clearly outlined the study objectives, potential risks, and benefits and assured the anonymity of the data. This study adheres to the provisions of the Ethical Code for Clinical and Counseling Psychology (2nd Edition) of the Chinese Psychological Society (2018) regarding the waiver of written informed consent. Given that the study uses anonymous online questionnaires and involves no potential risks, informed consent was obtained through participants clicking ‘I consent’ electronically, as approved by the Institutional Review Board. All the procedures involving human participants complied with the ethical standards of our institution.

SPSS 21.0 and AMOS 21.0 were used for data processing and statistical analysis. Participants (*N* = 669) were randomly divided into Sample 1 (*n* = 328) and Sample 2 (*n* = 341). The t-test was used to verify the balance of demographic characteristics after grouping. *T*-tests showed no significant differences in demographics (age, gender, only-child status, etc.) between groups (*p* > 0.05, Table 2), confirming balanced allocation. Sample 1 (*n* = 328) for exploratory factor analysis (EFA) and reliability and criterion-related validity tests and Sample 2 (*n* = 341) for confirmatory factor analysis (CFA). Pearson’s correlation analysis was used to investigate the relationship between the break-up distress and criterion variables. Internal consistency and split-half reliability tests were performed on the entire sample (*N* = 669).

**Table 2:** *t*-test of samples 1 and 2.

Variable	Group	Sample 1 (*n* = 328)	Sample 2 (*n* = 341)	*t*	*p*
Age	–	22.33 ± 3.29	22.44 ± 5.30	−0.33	0.744
Gender	Male	139	145	0.04	0.970
	Female	189	196		
Only children or not	Only children	128	157	1.84	0.067
	Not only children	200	184		
Breakup initiator	Self	102	101	−0.42	0.673
	Partner	93	78		
	Both	55	75		
	Neither	33	57		
	Unclear	45	30		
Number of dating relationships	Once	111	95	−0.39	0.701
	Twice	93	111		
	Three times	46	69		
	Four or more times	78	66		

## 3 Results

### 3.1 Project analysis

In this study, the critical ratio (CR) was selected as an indicator of item discriminability. The total scores were sorted from high to low, and the top 27% (scores above 50) and bottom 27% (scores below 22) were taken as the high- (*n* = 196) and low-score groups (*n* = 183), respectively, to test the differences between the high- and low-score groups for each item. If *t* was less than 3, the item was considered to have poor discriminability, and it might have been necessary to delete the item. The results of the independent samples *t*-test showed significant differences between the high- and low-score groups for all items (*p* < 0.01).

A homogeneity test was conducted to explore the relationship between each item and the total scale. If the item-total correlation coefficient is less than 0.4, the item is considered to have a low correlation with the overall construct of the scale, and deleting the item may be necessary. Results showed that the correlation coefficient *r* between the scores of each item and the total score was above 0.73 and reached a significant level (*p* < 0.01). Table 3 shows the item-total correlations of the Chinese version of the BDS as well as the results of the independent samples *t*-test for the scores of the high- and low-score groups on each item.

**Table 3:** Results of the project analysis (*n* = 669).

Item	*t*	*R*	*M*	*SD*	*Sk*	*Ku*
1	−36.599^**^	0.821^**^	2.08	1.08	0.26	−0.90
2	−32.240^**^	0.773^**^	2.17	1.14	0.26	−1.26
3	−43.844^**^	0.838^**^	1.88	1.11	−0.27	−0.61
4	−28.593^**^	0.734^**^	2.46	1.13	0.10	−1.38
5	−36.132^**^	0.802^**^	1.77	1.01	−0.34	0.03
6	−42.525^**^	0.836^**^	1.77	1.05	−0.44	−0.21
7	−35.563^**^	0.819^**^	1.77	1.04	−0.41	−0.06
8	−28.417^**^	0.739^**^	2.03	1.09	0.08	−0.98
9	−38.481^**^	0.788^**^	1.84	1.07	−0.29	−0.48
10	−46.344^**^	0.870^**^	1.95	1.04	0.03	−0.63
11	−37.533^**^	0.777^**^	2.00	1.16	−0.13	−1.04
12	−48.265^**^	0.863^**^	1.68	1.01	−0.62	0.48
13	−40.084^**^	0.814^**^	1.69	0.97	−0.51	0.50
14	−31.690^**^	0.760^**^	1.52	0.94	−1.06	1.55
15	−38.464^**^	0.808^**^	2.00	1.12	−0.03	−0.96
16	−38.695^**^	0.823^**^	1.88	1.09	−0.23	−0.58

^**^p < 0.01.

### 3.2 EFA

First, data were subjected to the Kaiser–Meyer–Olkin (KMO) and Bartlett’s sphericity tests. The KMO test was used to measure the partial correlation between variables, ranging from 0 to 1. Generally, when the KMO value is greater than 0.6, data are considered suitable for factor analysis. The Bartlett’s test of sphericity is used to test whether the correlation matrix is an identity matrix. If the test results were significant, the data were considered suitable for factor analysis. In the current study, the sample data were shown to be highly suitable for factor analysis, as the Bartlett’s test of sphericity value obtained was 9025.25 (*p* < 0.01), and the KMO value was 0.972. The sample data were then randomly divided into two groups: Sample 1 (*n* = 328) for EFA and Sample 2 (*n* = 341) for CFA.

The principal component analysis method was used to extract factors from the sample data, with eigenvalue ≥1 and absolute value of factor load ≥0.4 as extraction conditions. Results showed that only one factor had an eigenvalue greater than 1, and its explanatory variance was 64.81%. This results indicated that most of the variations in the data could be explained by this factor and that it possessed a relatively high explanatory power. The factor loadings of each item ranged from 0.567 to 0.900, indicating that the initial scale revision achieved good results. A strong correlation was found between each item and the extracted factors, and the scale effectively measured break-up distress (See Table 4 for further details).

**Table 4:** Results of the factor load of each item.

Item	Factor load	Item	Factor load
1	0.830	9	0.701
2	0.789	10	0.900
3	0.832	11	0.724
4	0.742	12	0.842
5	0.736	13	0.721
6	0.796	14	0.567
7	0.760	15	0.760
8	0.631	16	0.794

### 3.3 CFA

A CFA was conducted on Sample 2 data using the maximum likelihood estimation method via AMOS. Traditional fit indices were selected as the standard for model fitting: the ratio of the chi-square to degrees of freedom (*χ*^2^/*df*), goodness-of-fit index (GFI), adjusted goodness-of-fit index (AGFI), normalized fit index (NFI), and root mean square error of approximation (RMSEA). Among them, a *χ*^2^/*df* ratio less than 3, an absolute fit index RMSEA not greater than 0.08, GFI and other indices not less than 0.90, and an incremental fit index NFI not less than 0.90 indicate a good model fit. The CFA results in the current study indicated that all fit indices met the above criteria (*χ*^2^/*df* = 1.792, GFI = 0.935, AGFI = 0.915, NFI = 0.958, and RMSEA = 0.048). This demonstrates that the model has a good fit. In other words, the structure of the Chinese version of the BDS was in high-level agreement with the actual data and effectively reflected negative emotions and behavioral problems after the dissolution of a romantic relationship.

### 3.4 Reliability test

Tests were conducted using the Chinese version of the BDS to comprehensively evaluate its reliability. First, the internal consistency reliability test was performed, and Cronbach’s *α* was adopted as the measurement index. A value of Cronbach’s α closer to 1 suggests a stronger correlation among the various items of the scale, indicating a higher level of internal consistency of the scale. In the current study, Cronbach’s α of the Chinese version of the BDS was 0.96, indicating that the scale had extremely high internal consistency, and each item could measure the same concept well.

Second, a split-half reliability test was conducted. Split-half reliability was used to divide the scale items into two halves and to calculate the correlation between the scores of these two halves to evaluate the reliability of the scale. In the present study, the split-half reliability (*r_hh_*) of the Chinese version of the BDS was 0.96. Subsequently, it was corrected using the Spearman-Brown formula, and the corrected *r_xx_* value was 0.98. Both homogeneity reliability and split-half reliability reached good levels, which was above 0.9. This result demonstrated that the scale had good reliability and could measure the degree of break-up distress among college students in a relatively stable manner.

### 3.5 Criterion-related validity

In the present study, to test the criterion-related validity of the scale, correlations between the total BDS score and criterion variables such as depression, anxiety, and sleep were examined. Pearson correlation analysis showed that the total score on the BDS was significantly and positively correlated with depression (*r* = 0.55, *p* < 0.001), anxiety (*r* = 0.54, *p* < 0.001), and sleep (*r* = 0.47, *p* < 0.001). This result was consistent with that of the previous studies (Brassard et al., 2018; Field et al., 2009), indicating that individuals experiencing a higher level of break-up distress would have more severe negative emotions, such as depression and anxiety, and were also more likely to have sleep problems. This further verifies that the BDS effectively measures the degree of break-up distress among college students. Moreover, a reasonable association was found between the content measured using this scale and other psychological variables, demonstrating good criterion-related validity.

### 3.6 Differential testing

The results of the independent samples t-test show that males have higher break-up distress than females ([39.6 ± 14.9] *vs.* [33.6 ± 9.8], *t* = 5.17, *p* < 0.001). The study also investigated whether the participants developed a new romantic relationship after the breakup. Among them, 142 reported having developed a new relationship, 331 reported not having developed one, and 196 reported being unwilling to disclose. The current study only conducted a difference test for the “yes, I did” and “no, I did not” options. Results showed that participants who did not develop new relationships experienced more distress than those who did ([38.9 ± 15.1] *vs.* [33.4 ± 15.2], *t* = −3.65, *p* < 0.001).

In the online survey, the participants were presented with a question regarding the initiator of their most recent breakup, which included five response options: self, partner, both, neither, and unclear. The current study only conducted a difference test for the “self” and “partner” options. Results indicated that participants whose breakup was initiated by the partner (*n* = 186) had higher scores than those who initiated the breakup themselves (*n* = 188) ([41.1 ± 14.5] *vs*. [30.7 ± 13.8], *t* = 7.09, *p* < 0.001).

### 3.7 Measurement invariance

In the absence of measurement invariance testing, group differences derived from *t*-test analyses may emanate from genuine variations in latent constructs or divergent interpretations of measurement items across subgroups. Measurement invariance serves as a critical methodological framework for precluding the latter source of bias, ensuring that a scale measures the same underlying construct equally across groups. To fortify the robustness of the *t*-test inferences, a systematic approach to measurement invariance was implemented, comprising a baseline model specification, hierarchical parameter constraints, and nested model comparisons. (a) Weak invariance: Factor loadings were constrained to be invariant across groups while allowing intercepts and error variances to be freely estimated, thereby evaluating whether measurement indicators maintained consistent predictive validity for latent variables. (b) Strong invariance: Intercepts were additionally constrained to be equal to assess the equivalence of the indicator baseline levels across subgroups. (c) Strict invariance: A comprehensive constraint was simultaneously applied to the factor loadings, intercepts, and error variances to examine any significant discrepancies in factor structural equivalence and item-level measurement precision. Finally, the measurement invariance based on the fit indices of each model were determined. Generally, if CFI, NFI, and RMSEA are less than 0.03, the measurement invariance of the scale holds (Cheung and Rensvold, 2002). In this study, Gender and breakup initiators were used as representative demographic variables to test the invariance of the BDS.

Results show that the model fit indices all met the measurement requirements, proving that the configural invariance of the BDS across gender and breakup initiators holds. By further comparing the nested models, CFI, NFI, and RMSEA were all less than 0.01, indicating that the equivalence models of break-up distress across gender and breakup initiator were all valid, and the multiple-group comparison based on gender and break-up initiator had measurement significance. Thus, the psychological constructs measured by the BDS were consistent across genders and breakup initiators (see Tables 5, 6 for further details). This finding further indicates that the differences in break-up distress levels across genders and breakup initiators stem from actual psychological state disparities rather than scale bias.

**Table 5:** Results of the measurement invariance of the BDS across genders.

Model	CFI	NFI	RMSEA (90% CI)
Configural invariance	0.927	0.906	0.069 (0.065–0.074)
Weak invariance	0.927	0.905	0.067 (0.062–0.071)
Strong invariance	0.927	0.905	0.067 (0.062–0.071)
Strict invariance	0.927	0.905	0.067 (0.062–0.071)

**Table 6:** Results of the measurement invariance of the BDS across breakup initiators.

Model	CFI	NFI	RMSEA (90% CI)
Configural invariance	0.909	0.851	0.045 (0.042–0.048)
Weak invariance	0.909	0.850	0.044 (0.041–0.047)
Strong invariance	0.910	0.850	0.044 (0.041–0.047)
Strict invariance	0.909	0.850	0.045 (0.042–0.048)

## 4 Discussion

Break-up distress has emerged as a focal concern in psychological counseling for college students, with its profound implications for emotional and social well-being. Elevated levels of break-up distress are associated with a constellation of maladaptive responses, including but not limited to anxiety, depressive symptomatology, obsessive rumination on the breakup experience, attenuated interpersonal trust, compromised social competence, and somnological disturbances such as insomnia and nocturnal awakenings. These sequelae find theoretical grounding in attachment theory (Bowlby, 1980), which posits that the loss of intimacy in romantic partnerships precipitates the activation of anxious attachment schemas, thereby instigating compulsive cognitive rehashing of the breakup and protracted negative affect. This theoretical framework elucidates the observed social trust deficits in participants with higher BDS scores, as disrupted attachment bonds are conjectured to erode the foundational sense of interpersonal security (Brassard et al., 2018). In light of the deleterious effects of break-up distress on collegiate mental health, the present study undertakes the adaptation and validation of the Chinese version of the BDS, aspiring to furnish a robust psychometric instrument for researching romantic relationship termination within this demographic.

The results of the current study showed that the item-total correlations of the Chinese version of the BDS were significant, with correlation coefficients above 0.73. There were significant differences between the high- and low-score groups for each item, indicating that the items on the BDS had good discriminative power. The results of the EFA supported the one-dimensional model of the original scale. The results of the CFA showed that all model fit indices met the measurement requirements, indicating that the model fit well. Furthermore, the Chinese version of the BDS adopted the factor structure of the original scale, suggesting that the painful experiences of college students after the dissolution of romantic relationships showed cross-cultural consistency. In the Chinese culture, collectivist values emphasize the harmony and stability of interpersonal relationships. This may cause college students, when facing a breakup, to not only experience personal emotional distress but also worry about what others think, giving rise to additional psychological pressure. Items associated with social-relational impacts in the scale resonated well with the Chinese context (e.g., since the breakup, it has been hard for me to trust people), indicating its validity in measuring break-up distress among Chinese samples.

The single-factor structure of the original scale had a theoretical basis. Break-up distress is a multidimensional construct of cognitive-emotional-behavioral dysfunction following romantic relationship termination, characterized by obsessive preoccupation with the relationship and breakup, sustained negative emotional activation (e.g., distress and anger), and temporary social functional deficits (e.g., reduced interpersonal trust). Therefore, it is reasonable to measure this using a single-factor structure. Moreover, previous studies using the English version of the scale have verified that its single-factor structure has good reliability and validity (Field et al., 2009). This finding indicates that the structure can effectively measure the degree of break-up distress among college students, providing strong empirical evidence for its adoption in the Chinese version of the scale.

Previous research has shown that the longer the time since a breakup, the less the break-up distress experienced by participants (Field et al., 2009). Given the impact of time on break-up distress, the score of break-up distress on the second repeated measurement was lower than the first score, thus affecting test–retest reliability. Therefore, the current study did not conduct a test–retest reliability analysis. Results of the reliability analysis indicated that the Chinese version of the BDS had good internal consistency and split-half reliability.

The validity analysis in the present study showed that the total BDS score was significantly correlated with depression, anxiety, and sleep, which was consistent with existing research results. That is, break-up distress was significantly positively correlated with negative emotions such as depression, anxiety, guilt, and anger; and individuals with higher levels of break-up distress reported more sleep problems (Boelen and Reijntjes, 2009; Ford and Kamerow, 1989). As a painful experience for college students, the effects of breakups can be significant (Zhu et al., 2016).

The analysis of gender differences and the initiator of breakup revealed that males reported higher levels of break-up distress than females, which was consistent with the research conducted by Knox et al. (2000), Geng et al. (2023), and Zeng et al. (2022). For males who were less aware of issues in their intimate relationships, the breakup was more abrupt. The sudden change and difficulty in letting go of the pain lead to a higher level of break-up distress experienced by males (Hill et al., 1976; Oliffe et al., 2022; Sharp et al., 2023). Participants whose partners initiated the breakup (i.e., those who passively accepted the breakup) experienced more intense break-up distress than those who initiated the breakup themselves. Research has also shown that when a partner initiates a breakup, that is, when an individual passively ends a romantic relationship, they often experience more negative emotions such as anger and feelings of betrayal (Chen et al., 2016; Field et al., 2009; Moroz et al., 2018).

After confirming that the Chinese version of the BDS had good reliability and validity, this study further examined the measurement invariance between gender and breakup initiators. The Chinese version of the BDS showed strict equivalence across genders, indicating that the conceptual structure and measurement errors of break-up distress were equal between males and females. The differences in scores between males and females could directly reflect the differences in their levels of break-up distress. The Chinese version of the BDS met strict equivalence across breakup initiators, and this result ensured the effectiveness of comparing the scores of the different breakup initiators in subsequent studies.

This study contributes significantly to theoretical and practical applications. As documented in *The 2024 Report on the Mental Health Status of College Students*^1^ issued by *the Institute of Psychology, Chinese Academy of Sciences*, break-up distress has emerged as a pressing concern among Chinese undergraduates, and stable romantic relationships can potentially serve as a buffer to psychological vulnerabilities. However, the absence of a validated and reliable measurement tool for assessing break-up distress among Chinese college students has posed a substantial challenge to research endeavors and intervention efforts in this domain. This study focused on the adaptation and validation of the Chinese version of the BDS. The validated BDS can be integrated into university mental health screening programs, allowing counselors to identify students at risk of severe breakup distress early. For example, students with BDS scores above the 75th percentile could be referred to cognitive-behavioral therapy (CBT) groups to improve emotional regulation and rebuild interpersonal trust. The resultant instrument provides a standardized and empirically supported measure, thereby enabling a more rigorous investigation of the construct of break-up distress in the Chinese context. Notably, the CFA has revealed that the Chinese version of the scale adheres to a single-factor structure, which not only aligns with theoretical frameworks but also enhances its use in research and clinical settings. This structure renders the scale more concise and user-friendly for practical applications. For researchers, using a single-factor scale for data collection and analysis is convenient and efficient because there is no need to consider complex factor relationships. To ensure measurement validity, this concise structure can better meet the needs of research and practice.

However, this study has several limitations. First, Online self-report data may introduce selection bias, as participants who volunteered for the study might be more attentive to mental health compared to those who declined. Additionally, the lack of clinical interviews prevented us from verifying self-reported symptoms, potentially missing subtle psychological reactions. Future research could combine online surveys with in-person interviews or physiological measures (e.g., cortisol levels) to enhance data validity. Second, breakup time was not investigated in detail. Only participants who experienced a breakup in the past 6 months were selected. Future studies should accurately measure breakup time and combine multiple methods to explore its effect on the emotional development of college students. Third, when revising the scales, there might be cultural differences in understanding break-up distress. Although cultural differences were considered in the revision of the scale, there might still be some cultural factors that were not fully captured, which might affect the applicability of the scale to different cultural backgrounds. Future studies could also be combined with China’s collectivist culture, considering the sense of belonging and self-worth experienced after a breakup as the starting point for developing a local measurement tool for break-up distress. Finally, a limitation of this study is the lack of discriminant validity assessment. The absence of measuring and analyzing concepts unrelated to the core variables led to an incomplete validation of the measurement tool’s effectiveness. In future research, the study design should be redesigned to incorporate theoretically unrelated variables, and discriminant validity should be tested using methods such as correlation analysis to further improve the evaluation system for the effectiveness of measurement tools.

In summary, the Chinese version of the BDS has good reliability, validity, and discriminative power and can be used to assess the break-up distress perceived by college students in China.

## Data Availability

The raw data supporting the conclusions of this article will be made available by the authors, without undue reservation.

## Footnotes

^1^ https://www.psy.com.cn/article/view.asp?id=70219

## Appendix

### The Chinese version of Break-up Distress Scale

1. 我总会想起那个人以致于无法像平常一样做事。
2. 关于那个人的回忆让我心烦意乱。
3. 我感觉不能接受所经历的分手。
4. 我对和那个人有关的场景或经历念念不忘。
5. 我会忍不住对分手感到愤怒。
6. 我对分手感到难以置信。
7. 我对分手感到震惊或茫然。
8. 自从分手以后, 我很难去信任他人。
9. 自从分手以后, 我感觉自己失去了关心他人的能力, 或感觉对自己关心的人疏远了很多。
10. 自从分手以后, 我一直都感觉痛苦。
11. 我竭尽全力回避能让我想起那个人的东西。
12. 我觉得没有那个人, 生活是空虚的。
13. 我对这次分手感到怨恨。
14. 我对那些没有经历过这样分手的人感到嫉妒。
15. 自从分手以后, 我总是感到孤独。
16. 每当想起那个人时, 我会想哭。

### The English version of Break-up Distress Scale

1. I think about this person so much that it’s hard for me to do things I normally do.
2. Memories of the person upset me.
3. I feel I cannot accept the breakup I’ve experienced.
4. I feel drawn to places and things associated with the person.
5. I cannot help feeling angry about the breakup.
6. I feel disbelief over what happened.
7. I feel stunned or dazed over what happened.
8. Ever since the breakup it is hard for me to trust people.
9. Ever since the breakup I feel like I have lost the ability to care about other people or I feel distant from people I care about.
10. I have been experiencing pain since the breakup.
11. I go out of my way to avoid reminders of the person.
12. I feel that life is empty without the person.
13. I feel bitter over this breakup.
14. I feel envious of others who have not experienced a breakup like this.
15. I feel lonely a great deal of the time since the breakup.
16. I feel like crying when I think about the person.

## References

[ref1] Boelen P. A. Den Bout J. V. De Keijser J. O. S. Hoijtink H. (2003). Reliability and validity of the Dutch version of the inventory of traumatic grief (ITG). Death Stud. 27, 227–247. doi: 10.1080/07481180302889, PMID: 12703504

[ref2] Boelen P. A. Reijntjes A. (2009). Negative cognitions in emotional problems following romantic relationship break-ups. Stress. Health 25, 11–19. doi: 10.1002/smi.1219

[ref3] Bowlby J. (1980). “Loss, sadness and depression” in Attachment and loss, vol. 3 (New York: Basic Books).

[ref4] Brassard A. St-Laurent Dube M. Gehl K. Lecomte T. (2018). Romantic Attachment and Post Breakup Depression Symptoms and Suicidal Behaviour. [Attachement amoureux, symptomes depressifs et comportements suicidaires en contexte de rupture amoureuse.]. Sante Ment. Que. 43, 145–162. Available at: https://lib.umslib.v.zzu.edu.cn/https/vpn/1660/P7TXE55GPNSXT3LPMNTT6Z5MMF3GT7UBPSTT6Z5P/wos/alldb/full-record/MEDLINE:32338700 32338700

[ref5] Brewer G. Abell L. (2017). Machiavellianism and romantic relationship dissolution. Pers. Individ. Differ. 106, 226–230. doi: 10.1016/j.paid.2016.11.001

[ref6] Brislin R. W. (1970). Back-translation for cross-cultural research. J. Cross-Cult. Psychol. 1, 185–216. doi: 10.1177/135910457000100301

[ref7] Bronfman G. Ladd-Luthringshauser H. Goodman L. R. Sockol L. E. (2016). Predictors of breakup distress among residential college students. Coll. Stud. Aff. J. 34, 3–12. doi: 10.1353/csj.2016.0015

[ref8] Brüning M. (2022). Separations of romantic relationships are experienced differently by initiators and noninitiators. Proc. Natl. Acad. Sci. USA 119:e2020901119. doi: 10.1073/pnas.2020901119, PMID: 35648824 PMC9191343

[ref9] Chen S. Moroz S. Daljeet K. (2016). The dark triad and breakup distress: indirect effects through relationship investment and commitment. Pers. Individ. Differ. 101:471. doi: 10.1016/j.paid.2016.05.106

[ref10] Cheung G. W. Rensvold R. B. (2002). Evaluating goodness-of-fit indexes for testing measurement invariance. Struct. Equ. Model. 9, 233–255. doi: 10.1207/S15328007SEM0902_5

[ref11] Cui S. Zhou B. Liu D. Xiong Z. Zhang L. Qiu Z. (2014). SCL-90 psychological symptom checklist used in overseas projects. J. Saf. Sci. Technol. 10, 61–67. doi: 10.11731/j.issn.1673-193x.2014.z2.014

[ref12] Dooley B. Fitzgerald A. Giollabhui N. M. (2015). The risk and protective factors associated with depression and anxiety in a national sample of Irish adolescents. Ir. J. Psychol. Med. 32, 93–105. doi: 10.1017/ipm.2014.83, PMID: 30185277

[ref13] Duan X. Cheng J. (2006). Psychological crisis intervention for college students. Beijing: Science Press, 21–24.

[ref14] Field T. Diego M. Pelaez M. Deeds O. Delgado J. (2009). Breakup distress in university students. Adolescence 44, 705–727. doi: 10.1016/j.addbeh.2007.07.016 20432597

[ref15] Field T. Diego M. Pelaez M. Deeds O. Delgado J. (2010). Breakup distress and loss of intimacy in university students. Psychology 1, 173–177. doi: 10.4236/psych.2010.13023

[ref16] Ford D. E. Kamerow D. B. (1989). Epidemiologic study of sleep disturbances and psychiatric disorders: an opportunity for prevention? JAMA 262, 1479–1484. doi: 10.1001/jama.262.11.1479, PMID: 2769898

[ref17] Geng Y. G. Zhan T. T. Zhang Y. W. Shi L. P. Yu J. J. Jin W. J. (2023). Why don’t you tell me? The mediating role of self-concealment in the relationship between Machiavellianism and break-up distress. Curr. Psychol. 42, 17000–17007. doi: 10.1007/s12144-022-02911-8

[ref18] Harake N. R. Dunlop W. L. (2020). Storying the heartbreak the transformational processing of romantic breakups. Narrat. Inq. 30, 18–40. doi: 10.1075/ni.18064.har

[ref19] Hill C. T. Rubin Z. Peplau L. A. (1976). Breakups before marriage: the end of 103 affairs. J. Soc. Issues 32, 147–168. doi: 10.1111/j.1540-4560.1976.tb02485.x

[ref20] Huang H. Q. Zheng L. (2012). Analysis and reflection on college students’ views on love from the perspective of culture. Beijing Educ. 9, 26–28. Available at: https://kns.cnki.net/kcms2/article/abstract?v=C4JADW50D8uT2D1pCNhqzzegI7rI3ktob5-gS0o3Wwtjt-19lI18Nc5WWGyAwmlru53T35NQOlNsrzTrgl6338CDanqkKj2lsWPwUhpRud_wlYDTggxoPLzR78yWCxI3bgxRrIkMLxyfeVbN8THqXCLrBvTt1_oz8SMrRc2cePTI92MSId-TYA==&uniplatform=NZKPT&language=CHS

[ref21] Johnson M. D. Lavner J. A. Stanley S. M. Rhoades G. K. (2024). Gender differences-or the lack thereof-in the prediction of relationship dissolution among unmarried mixed-gender couples from the United States. J. Soc. Pers. Relat. 41, 3316–3336. doi: 10.1177/02654075241265063

[ref22] Knox D. Zusman M. Kaluzny M. Cooper C. (2000). College student recovery from a broken heart. Coll. Stud. J. 34:322.

[ref23] Moroz S. Chen S. Daljeet K. N. Campbell L. (2018). The dark triad and break-up distress. Pers. Individ. Differ. 132, 52–59. doi: 10.1016/j.paid.2018.05.022

[ref24] Narrow W. E. Clarke D. E. Kuramoto S. J. Kraemer H. C. Kupfer D. J. Greiner L.. (2013). DSM-5 field trials in the United States and Canada, part III: development and reliability testing of a cross-cutting symptom assessment for DSM-5. Am. J. Psychiatry 170, 71–82. doi: 10.1176/appi.ajp.2012.12071000, PMID: 23111499

[ref25] Norona J. C. Scharf M. Welsh D. P. Shulman S. (2018). Predicting post-breakup distress and growth in emerging adulthood: the roles of relationship satisfaction and emotion regulation. J. Adolesc. 63, 191–193. doi: 10.1016/j.adolescence.2018.01.001, PMID: 29328956

[ref26] Oliffe J. L. Kelly M. T. Montaner G. G. Seidler Z. E. Ogrodniczuk J. S. Rice S. M. (2022). Masculinity and mental illness in and after men’s intimate partner relationships. Ssm-Qualitative Res. Health 2:100071. doi: 10.1016/j.ssmqr.2022.100039

[ref27] Prigerson H. G. Maciejewski P. K. Reynolds C. F. 3rd Bierhals A. J. Newsom J. T. Fasiczka A.. (1995). Inventory of complicated grief: a scale to measure maladaptive symptoms of loss. Psychiatry Res. 59, 65–79. doi: 10.1016/0165-1781(95)02757-2, PMID: 8771222

[ref28] Rhoades G. K. Kamp Dush C. M. Atkins D. C. Stanley S. M. Markman H. J. (2011). Breaking up is hard to do: the impact of unmarried relationship dissolution on mental health and life satisfaction. J. Fam. Psychol. 25, 366–374. doi: 10.1037/a0023627, PMID: 21517174 PMC3115386

[ref29] Sánchez-Porro D. Silva-Vicuña y. J. (2024). Género y rol iniciador en las rupturas sentimentales: diferencias en el afrontamiento. Revista de Psicología 42, 870–899. doi: 10.18800/psico.202402.008

[ref30] Sharp P. Ogrodniczuk J. S. Sha M. T. Kelly M. T. Montaner G. G. Kealy D.. (2023). Working with men in the context of distressed and disrupted intimate partner relationships: a qualitative study. Patient Educ. Couns. 115:107928. doi: 10.1016/j.pec.2023.107873, PMID: 37421685

[ref31] Sprecher S. (1994). Two sides to the breakup of dating relationships. Pers. Relat. 1, 199–222. doi: 10.1111/j.1475-6811.1994.tb00062.x

[ref32] Wang X. Zhang R. (2011). Affection of broken-up on college students’ love attitude and psychological health. Chin. J. Health Psychol. 19, 588–600. doi: 10.13342/j.cnki.cjhp.2011.05.016

[ref33] Xiao Y. Xiaoming J. (2018). A qualitative research of grief process after lovelorn in college students. Chin. Ment. Health J. 32, 233–238. doi: 10.3969/j.issn.1000-6729.2018.03.009

[ref34] Zeng P. Y. Jin W. J. Shi Y. B. Hu W. Y. Geng Y. G. Zhan T. T. (2022). Struggling or liberating? The effects of Machiavellianism on break-up distress. Int. J. Environ. Res. Public Health 19:14016. doi: 10.3390/ijerph192114581, PMID: 36361460 PMC9656552

[ref35] Zhang A. L. Yu R. P. Gong Y. J. (2023). The influence of traditional culture on college students’ views on love: taking classical poetry as an example. Culture J. 2, 182–185. Available at: https://kns.cnki.net/kcms2/article/abstract?v=C4JADW50D8sM2_k2DemBkud3ln5r3d-r6irp_qqwZEcwvha4BPsa0B3-Xj7-5ToziYvvfs2BIh-Ru9t2Yhh6onh6jNojuLsxMy8d3PlKWoxgiwGqZDhji3m-R_wqYo5tGZTyWSY5suq_NL9TW0n4AdYjVeHzBxrnpeQmQvGu1Ub_2vhwiUW_HQ==&uniplatform=NZKPT&language=CHS

[ref36] Zhu X. L. Geng Y. G. Shi L. P. Sai X. Y. (2019). Preliminary application of DSM-5 level 1 cross-cutting symptom measure-adult in college students. Chin. J. Sch. Health 40, 1909–1911. doi: 10.16835/j.cnki.1000-9817.2019.12.042

[ref37] Zhu H. Song Z. Deng Y. Xiong Y. Chen R. (2016). Comparing different attributions of the breaking-up of college romance from different positions. Psychol Tech. App. 4, 154–159. doi: 10.16842/j.cnki.issn2095-5588.2016.03.006

